# Effect of tofacitinib therapy on angiotensin converting enzyme activity in rheumatoid arthritis

**DOI:** 10.3389/fmed.2023.1226760

**Published:** 2023-10-09

**Authors:** Dorottya Kacsándi, Miklós Fagyas, Ágnes Horváth, Edit Végh, Anita Pusztai, Monika Czókolyová, Boglárka Soós, Attila Ádám Szabó, Attila Hamar, Zsófia Pethő, Nóra Bodnár, György Kerekes, Katalin Hodosi, Szilvia Szamosi, Gabriella Szűcs, Zoltán Papp, Zoltán Szekanecz

**Affiliations:** ^1^Department of Rheumatology, Faculty of Medicine, University of Debrecen, Debrecen, Hungary; ^2^Division of Clinical Physiology, Department of Cardiology, Faculty of Medicine, University of Debrecen, Debrecen, Hungary; ^3^Kálmán Laki Doctoral School of Biomedical and Clinical Sciences, University of Debrecen, Debrecen, Hungary; ^4^Intensive Care Unit, Department of Medicine, Faculty of Medicine, University of Debrecen, Debrecen, Hungary

**Keywords:** rheumatoid arthritis, JAK inhibition, tofacitinib, angiotensin converting enzyme, vascular disease, targeted therapy

## Abstract

**Introduction:**

The Renin-Angiotensin-Aldosterone system (RAAS) has been implicated in the regulation of the cardiovascular system and linked to rheumatoid arthritis (RA). Little information has become available on the effects of Janus kinase (JAK) inhibition on RAAS. Here we studied the effects of 12-month tofacitinib treatment on angiotensin converting enzyme (ACE), ACE2 production and ACE/ACE2 ratios in RA along with numerous other biomarkers.

**Patients and methods:**

Thirty RA patients were treated with tofacitinib in this prospective study. Serum ACE concentrations were assessed by ELISA. ACE2 activity was determined by a specific quenched fluorescent substrate. ACE/ACE2 ratios were calculated. We also determined common carotid intima-media thickness (ccIMT), brachial artery flow-mediated vasodilation (FMD) and carotid-femoral pulse-wave velocity (cfPWV) by ultrasound. C-reactive protein (CRP), rheumatoid factor (RF) and anti-citrullinated protein autoantibodies (ACPA) were also determined. All measurements were performed at baseline, as well as after 6 and 12 months of tofacitinib treatment.

**Results:**

After the dropout of 4 patients, 26 completed the study. Tofacitinib treatment increased ACE levels after 6 and 12 months, while ACE2 activity only transiently increased at 6 months. The ACE/ACE2 ratio increased after 1 year of therapy (*p* < 0.05). Logistic regression analyses identified correlations between ACE, ACE2 or ACE/ACE2 ratios and RF at various time points. Baseline disease duration also correlated with erythrocyte sedimentation rate (ESR) (*p* < 0.05). One-year changes of ACE or ACE2 were determined by tofacitinib treatment plus ACPA or RF, respectively (*p* < 0.05).

**Conclusion:**

JAK inhibition increases serum ACE and ACE/ACE2 ratio in RA. Baseline inflammation (ESR), disease duration and ACPA, as well as RF levels at various time points can be coupled to the regulation of ACE/ACE2 ratio. The effect of tofacitinib on RAAS provides a plausible explanation for the cardiovascular effects of JAK inhibition in RA.

## Introduction

Janus kinases (JAK) are involved in the signaling of multiple cytokines (1, 2). Various JAKs are involved in the inflammatory process underlying rheumatoid arthritis (RA) (3, 4). Up to now, four JAK inhibitors (JAKi), tofacitinib, baricitinib, upadacitinib and filgotinib have been approved for the treatment of RA (5).

RA has been associated with increased cardiovascular (CV) morbidity and mortality (6–10). Abnormal CV pathophysiology can be observed very early, even in RA patients without clinical atherosclerotic CV disease (ASCVD) (6, 10, 11). Ultrasound-based imaging is suitable to detect preclinical vascular pathophysiology (12). Endothelial dysfunction of the brachial artery, carotid atherosclerosis and increased arterial stiffness are indicated by impaired endothelium-dependent, flow-mediated vasodilation (FMD), increased common carotid intima-media thickness (ccIMT) and carotid-femoral pulse-wave velocity (cfPWV), respectively (9, 12). These preclinical abnormalities might predict subsequent CV events in RA (12, 13).

Angiotensin-converting enzyme (ACE) is a member of the Renin-Angiotensin-Aldosterone system (RAAS). RAAS regulates blood pressure, as well as salt-water homeostasis (14). ACE promotes the conversion of angiotensin I to angiotensin II and also regulates bradykinin metabolism (14). ACE is involved in, among many other diseases, ASCVD, hypertension and heart failure (14, 15). ACE inhibitors are very frequently prescribed (14, 15). ACE2 is a homolog of ACE and exerts monocarboxy-peptidase activity (16). ACE2 stimulates the generation of angiotensin peptides Ang_1-9_ and Ang_1-7_ from Ang-I and Ang-II, respectively (16, 17). ACE2 has vasculoprotective and antihypertensive effects by counter-regulating RAAS (17, 18). Increased ACE2 activity has been found in advanced heart failure, hypertension and ventricular arrhythmias (17, 18).

ACE and ACE2 levels and activities are detectable in the serum. ACE, but not ACE2 activity is influenced by ACE inhibitors (15, 17). Changes in soluble ACE and ACE2 functions in the serum can be closely related to antiparallel changes of tissue activities of these enzymes (17, 19).

In addition to ASCVD and hypertension, ACE and ACE2 might also play a role in arthritides including RA. *ACE* gene insertion–deletion (I/D) polymorphism has been associated with RA. The DD genotype might enhance susceptibility to RA (20–22). Moreover, increased plasma Ang-II, Ang_1-7_ and ACE levels, ACE/ACE2 ratios, as well as decreased ACE2 release were found in RA. ACE2 levels showed negative correlation with ccIMT (23, 24). Moreover, there are increased synovial fluid ACE levels in RA in comparison to osteoarthritis (25, 26). Within the RA synovial tissue, synovial macrophages and endothelial cells express ACE (27).

There have been very few studies on the possible effects of disease modifying anti-rheumatic drugs (DMARDs), primarily biologic (bDMARDs) and targeted synthetic DMARDs (tsDMARDs) on ACE and ACE2. In one study, ACE2 levels were significantly decreased in RA patients treated with tumor necrosis factor (TNF)-inhibitors versus healthy controls (28). Recently, we found that anti-TNF agents increased ACE and ACE2 levels, as well as the ACE/ACE2 ratio in RA patients. We correlated ACE and ACE2 levels with disease duration, C-reactive protein (CRP), rheumatoid factor (RF), FMD and ccIMT (29).

The possible relationship between the RAAS system and JAK in inflammation is greatly unknown. In a study, He et al. (30) showed that RAAS might promote inflammation in human and murine colitis through the JAK2/STAT pathway, whereby an abundance of Ang-II in the colon supposedly promoted inflammation in the colon via JAK2/STAT1/3 signaling. In renin transgenic mice, tofacitinib inhibited the phosphorylation of JAK2 and STAT1/3 leading to the attenuation of colitis and better survival (30). We have not found any other studies on the possible effects of JAKi on RAAS/ACE in arthritis.

In summary, there has been little information on the effects of JAKi on ACE and ACE2 productions and on their correlation with disease-related, inflammatory and vascular biomarkers and vascular pathophysiology (FMD, ccIMT, cfPWV). We have recently studied an RA cohort where patients underwent tofacitinib therapy for 1 year. Moreover, we have previously reported tofacitinib effects on vascular pathophysiology and metabolism in this cohort (31, 32). Here we studied ACE and ACE2 in tofacitinib-treated RA patients. We also compared data on ACE and ACE2 with markers of inflammation, autoantibodies and markers of vascular pathophysiology in order to investigate the effects of JAKi on RAAS.

## Patients and methods

### Patients and study design

Altogether 30 patients with active RA were included in this prospective study. We applied the following inclusion criteria: definitive diagnosis of RA (33); high or moderate baseline disease activity [28-joint Disease Activity Score (DAS28) > 3.2], as well as the indication of targeted therapy. Patients were either targeted therapy-naïve (*n* = 16) or underwent an appropriate washout procedure after stopping biologics (*n* = 14). Exclusion criteria included non-RA inflammatory disorders; acute infection; any contraindication to tofacitinib therapy; uncontrolled chronic diseases (cardiovascular, renal, hepatic, malignant) within the past 10 years.

The 30 enrolled patients received either 5 mg or 10 mg tofacitinib twice daily (bid). Although only the 5 mg bid dose is registered in the EU, we also included 10 mg bid dose as the latter dose is used in many countries including the US and other non-EU countries. Tofacitinib was used in combination with a conventional synthetic DMARD [23 methotrexate (MTX), 7 leflunomide]. These DMARDs had been administered to the patients in stable doses at least 1 year prior to this study. The dose of these DMARDs were not modified throughout the study. Most patients had been treated with corticosteroids prior to the study, however, none of them had been on glucocorticoids for ≥3 months before and during the study.

Four patients (2 in the 5 mg bid and 2 in the 10 mg bid group) dropped out. This, 26 patients completed the study. Patient characteristics are presented in Table 1.

**Table 1:** Patient characteristics.

	Tofacitinib-treated patients
Number of patients (*n*)	26
female:male ratio	23:3
age (mean ± SD) (median), years	51.9 ± 9.7 (48)
BMI (mean ± SD) (median), kg/m^2^	30.3 ± 7.4 (34)
Positive CV history (*n*)	6
Positive history of hypertension (*n*)	13
Positive history of diabetes mellitus (*n*)	2
Smoking (current) (*n*)	7
disease duration (mean ± SD) (median), years	7.5 ± 4.8 (9.2)
RF positivity, *n* (%)	22 (84.6)
Anti-CCP positivity, *n* (%)	22 (84.6)
DAS28 (baseline) (mean ± SD) (median)	5.12 ± 0.82 (4.88)
ACE inhibitor treatment	8

ACE, angiotensin-converting enzyme; BMI, body mass index; CCP, anti-cyclic citrullinated peptide; CV, cardiovascular; DAS28, 28-joint disease activity score; RF, rheumatoid factor; SD, standard deviation.

The study was approved by the Hungarian Scientific Research Council Ethical Committee (approval No. 56953-0/2015-EKL). Written informed consent was obtained from each patient and assessments were carried out according to the Declaration of Helsinki and its amendments.

### Clinical assessment

Detailed medical history was taken first. We collected data on history of ASCVD, past or current smoking, chest pain, high blood pressure and diabetes mellitus by a questionnaire (Table 1). All clinical assessments were performed at baseline, as well as after 6 and 12 months of tofacitinib therapy.

### Assessment of vascular physiology by ultrasound

Ultrasound-based functional vascular assessments, such as brachial artery FMD, cfPWV and ccIMT measurements have been carried out at baseline and during the follow-up. Details of investigations were thoroughly described and published previously (32, 34). In the present study, FMD, ccIMT and cfPWV data are only used in the correlation analyses.

### Laboratory measurements and disease activity

Erythrocyte sedimentation rate (ESR) was determined by a standard procedure. Serum high sensitivity CRP (hsCRP; normal: ≤5 mg/L) and IgM RF (normal: ≤50 IU/mL) were assessed by quantitative nephelometry (Cobas Mira Plus, Roche Diagnostics, Basel, Switzerland), using CRP and RF reagents (both Dialab Ltd., Budapest, Hungary). Anti-citrullinated peptide (ACPA) autoantibodies were determined in the serum using Immunoscan-RA 2^nd^ generation cyclic citrullinated peptide (CCP2) ELISA test (Euro Diagnostica, Malmö, Sweden; normal: ≤ 25 IU/mL). We performed these assays according to the instructions of the manufacturer. In order to determined RA disease activity, 3-variable DAS28 (CRP) was calculated.

### Assessment of serum ACE concentration

Serum ACE levels were determined by a commercial human ACE ELISA (R&D Systems) as described previously (15, 29). ELISA plates (Greiner Bio-One) were coated with 80 ng/well capture antibody. The remaining binding sites were blocked using reagent diluent [10 mg/mL bovine serum albumin (Sigma-Aldrich) in Dulbecco’s phosphate buffered saline solution (PBS, Gibco)]. Diluted sera (100x) were added to the wells, and the antibody–antigen complexes were labeled by a biotinylated detection antibody (20 ng/well). Diluted (200x) streptavidin-conjugated horseradish-peroxidase was then added to the wells. Finally, the amounts of complexes were detected with a substrate solution containing 0.3 mg/mL tetramethylbenzidine, 0.1 mM H_2_O_2_ and 50 mM acetic acid. This reaction was terminated after 20 min by the addition of 0.5 M HCl, and the optical density (OD) was determined at 450 nm using a fluorescence microplate reader in absorbance mode (Clariostar; BMG Labtech GmbH, Offenburg, Germany). ACE levels are expressed as ng/mL.

### Determination of serum ACE2 activity

Serum ACE2 activity was evaluated using a specific quenched fluorescent substrate as previously described (17, 29). The reaction mixture (200 μL) contained 20 μL serum, 100 μM ACE2-specific fluorescent substrate (7-methoxycoumarin-4-yl)acetyl-Ala-Pro-Lys(2,4-dinitrophenyl)-OH; [Mca-APK(Dnp)] (Peptide 2.0, USA) 500 mM NaCl, 10 μM ZnCl_2_ in 75 mM TRIS HCl buffer, pH 6.5 [all from Sigma (St. Louis, MO, USA) if not indicated otherwise]. The reaction was carried out in black 96-well microtiter plates (Greiner Bio-One, Frickenhauser, Germany). The assay was continuously monitored by measuring the increase in fluorescence (λ_ex_ = 340 nm, λ_em_ = 405 nm) upon substrate hydrolysis using a fluorescence microplate reader (Clariostar; BMG Labtech GmbH, Offenburg, Germany). Initial enzyme activities were determined from the linear rate of fluorescence increase over the 10–120 min time course. The increase in fluorescence was plotted and fitted with a linear regression. Serum ACE2 activity was calculated by the following equation:


$$\mathrm{ACE}2\;\mathrm{activity}=\left(S/k\right)*D$$

S: rate of observed increase in fluorescence intensity;

k: change in fluorescence intensity upon the complete cleavage of 0.1 nmol of Mca-APK(Dnp);

D: dilution of the serum sample.

One unit of fluorescence (UF) corresponds to the quantity of enzyme which can degrade 0.1 nmol Mca-APK(Dnp) in 1 h at 37°C. Serum ACE2 activity assay specificity was tested by the specific human ACE2 inhibitor DX600 before (17). ACE2 activity is expressed in UF/mL units. ACE inhibitors do not influence ACE2 activity.

We also calculated the ratio of ACE concentration / ACE2 activity, as a good indicator of ACE and ACE2 redistribution (17, 19, 23) as we also reported before (29).

All assays were performed using the same lot and under the same standardized experimental conditions.

### Statistical analysis

Statistical analysis was conducted by SPSS 26.0 (IBM, Armonk, NY, USA) software. Data are expressed as the mean ± SD or percentages for continuous or categorical variables, respectively. We used Kolmogorov–Smirnov, two-tailed t and Wilcoxon tests to evaluate continuous variables. Nominal variables were compared using the χ^2^ or Fisher’s exact test, as appropriate. Simple correlations were determined by Pearson’s analysis. In addition, uni-and multivariable regression analyses using the stepwise method were used to determine independent associations between ACE levels or ACE2 activity (dependent variables) and other parameters (independent variables). The β coefficients showing linear correlations between two parameters were calculated. The B (+95% CI) regression coefficient showed independent associations between dependent and independent variables over time.

General linear model (GLM) repeated measures analysis of variance (RM-ANOVA) was conducted in order to evaluate the effects of various biomarkers in addition to tofacitinib treatment itself on one-year changes in ACE levels or ACE2 activity (dependent variable). In the RM-ANOVA analysis, partial η^2^ is given as an effect size indicator. Values of 0.01, 0.06 and 0.14 suggest small, medium and large effects, respectively. The power was estimated using the G*-Power 3 software (35). We considered *p* values <0.05 significant in all statistical tests.

The reliability of the vascular pathophysiology assessments tested by inter-item and intraclass correlation (ICC) before (9, 36). With respect to the FMD, ccIMT and cfPWV tests, ICC = 0.470; F-test value: 1.887; *p* = 0.001.

## Results

### Characteristics of patients

These data have been published before based on other studies emerging from the very same cohort (31, 32, 37). Eventually 26 patients completed the study (31, 32, 37). The characteristics of these 26 patients are included in Table 1.

### Effects of tofacitinib therapy on disease characteristics and vascular pathophysiology

As published before, one-year tofacitinib treatment was highly effective in controlling RA. Tofacitinib treatment significantly improved DAS28, CRP and Health Assessment Questionnaire (HAQ) both after 6 and 12 months (31, 32, 37). Regarding vascular function, in brief, FMD and cfPWV showed no changes, while ccIMT increased over time (32). In this study we only used the vascular ultrasonography data in order to correlate them with the ACE, ACE2 and ACE/ACE2 results. Thus, none of the data to be presented below have been published yet.

### Effects of JAKi on ACE levels, ACE2 activity and ACE/ACE2 ratio

**Figure 1:**
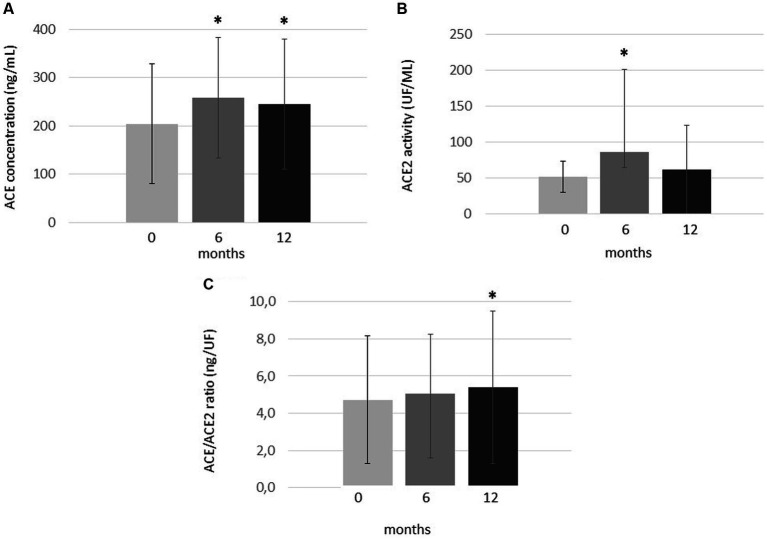
One-year changes of **(A)** ACE levels, **(B)** ACE2 activity and **(C)** ACE/ACE2 ratio upon one-year tofacitinib treatment. Data indicate mean ± SD. * Indicates significant differences compared to baseline (*p* < 0.05). Kolmogorov–Smirnov, two-tailed t and Wilcoxon tests were applied to evaluate differences between these continuous variables.

In this RA cohort, serum ACE levels significantly increased both after 6 (257.7 ± 125.0 ng/mL; *p* = 0.001) and 12 months of tofacitinib therapy (244.9 ± 135.2 ng/mL; *p* = 0.002) in comparison to baseline (204.2 ± 124.1 ng/mL; Figure 1A).

ACE2 activity significantly but transiently increased after 6 months (86.4 ± 115.2 UF/mL; *p* = 0.012) compared to baseline (51.7 ± 21.9 UF/ml). However, ACE2 activity then decreased and became similar to baseline after 12 months (61.5 ± 61.7 UF/ml; *p* = 0.328; Figure 1B).

Finally, in order to study the ACE/ACE2 balance, we calculated ratios of ACE concentrations and ACE2 activity (ACE/ACE2 ratios; Figure 1C). In our cohort, the ACE/ACE2 ratio was similar after 6 months (5.05 ± 3.18 ng/UF) and baseline (4.71 ± 3.44 ng/UF; *p* = 0.317). However, this ratio significantly increased after 12 months of treatment (5.39 ± 4.09 ng/UF) compared to baseline (*p* = 0.025; Figure 1C).

There have been no significant differences in ACE, ACE2 or ACE/ACE2 between the 5 mg bid and 10 mg bid tofacitinib doses (data are not shown). Also when comparing treatment-naïve patients (*n* = 14) with those after a washout period (*n* = 12), similar tendencies were observed with respect to ACE, ACE2 and ACE/ACE2 changes (data are not shown).

### Correlations of ACE levels, ACE2 activity and ACE/ACE2 ratio with parameters of RA

In the univariable regression analysis, ACE levels were independently and positively associated with RF concentrations and/or RF seropositivity at various time points (*p* < 0.05). In addition, ACE concentration at baseline also significantly correlated with ESR at baseline (*p* = 0.008; Table 2). Baseline ACE2 activity independently correlated with disease duration (*p* = 0.025). Moreover, ACE2 activity after 6 and 12 months of treatment were positively associated with RF concentrations at different time points (p < 0.05; Table 2). RF positivity was only associated with ACE levels but not with ACE2 activity (Table 2). ACE/ACE2 after 6 months of treatment positively correlated with baseline RF (*p* = 0.033). Baseline or 12-month ACE/ACE2 ratios did not show any correlations with any studied parameters. Among these associations, the multivariable regression analysis confirmed the correlations between baseline ACE levels and ESR (*p* = 0.008) and between ACE and RF concentrations after 12 months of therapy (*p* < 0.001; Table 2). We did not find any associations between ACE, ACE2 or ACE/ACE2 ratio and any of the imaging markers of vascular pathophysiology (FMD, ccIMT, cfPWV).

**Table 2:** Univariable and multivariable regression analysis of ACE and ACE2.

Dependent variable	Independent variable	Univariable analysis				Multivariable analysis			
		*β*	*p*	*B*	CI 95%	*β*	*p*	*B*	CI 95%
*ACE conc.-0*	*ESR-0*	0.510	0.008	2.968	0.857–5.080	0.510	0.008	2.968	0.857–5.080
	*RF-0*	0.601	0.001	0.340	0.149–0.530				
*ACE conc.-6*	*RF conc.-0*	0.605	0.001	0.345	0.153–0.536				
	*RF conc.-6*	0.753	<0.001	0.518	0.328–0.709				
	*RF pos.-0*	0.468	0.016	145.495	29,684–261,306				
*ACE conc.-12*	*RF conc.-0*	0.521	0.006	0.321	0.099–0.542				
	*RF conc.-6*	0.628	0.001	0.467	0.223–0.711				
	*RF conc.-12*	0.666	<0.001	0.512	0.271–0.754	0.666	<0.001	0.512	0.271–0.754
	*RF pos.-0*	0.497	0.010	167.318	44.408–290.228				
*ACE2 act.-0*	*DD-0*	0.439	0.025	1.833	0.251–3.416				
*ACE2 act.-6*	*RF conc.-0*	0.418	0.034	0.219	0.018–0.42				
*ACE2 act.-12*	*RF conc.-0*	0.670	<0.001	0.188	0.100–0.276				
	*RF conc.-6*	0.457	0.019	0.155	0.028–0.282				
	*RF conc.-12*	0.436	0.026	0.153	0.020–0.286				
*ACE/ACE2-0*	–								
*ACE/ACE2-6*	*RF conc.-0*	0.419	0.033	0					
*ACE/ACE2-12*	–								

The numbers 0, 6 and 12 represent baseline, 6-month and 12-month results. ACE, angiotensin-converting enzyme; act., activity; conc., concentration; DD, disease duration; ESR, erythrocyte sedimentation rate; pos., positivity; RF, rheumatoid factor.

Finally, RM-ANOVA analysis was conducted in order to assess determinants of ACE or ACE2 changes between baseline, 6 and 12 months. The one-year change in ACE concentration was determined by tofacitinib treatment together with ACPA seropositivity at baseline (*p* = 0.016). Moreover, the 12-month change in ACE2 activity was rather determined by JAKi effects together with RF concentration at baseline (*p* = 0.043; Table 3).

**Table 3:** Significant results of general linear model (GLM) repeated measures analysis of variance (RM-ANOVA) test determining the effects of treatment and other independent variables on ACE concentration or ACE2 activity as dependent variables.

Dependent variable	Effect	*F*	*p*	Partial η^2^
*ACE conc.-0–6-12*	ACPA pos.-0 * treatment	4.974	0.016	0.302
*ACE2 act.-0–6-12*	RF conc.-0 * treatment	4.174	0.043	0.148

The numbers 0, 6 and 12 represent baseline, 6-month and 12-month results. ACE, angiotensin-converting enzyme; ACPA, anti-citrullinated peptide antibody; act., activity; conc., concentration; pos., positivity; RF, rheumatoid factor.

## Discussion

This might be the first study on the effects of a JAKi on the ACE-ACE2 system. In our study, one-year tofacitinib therapy significantly increased ACE levels in RA. JAK inhibition also transiently augmented ACE2 activity. ACE/ACE2 ratios significantly increased after 12 months of treatment. Moreover, baseline, 6-and 12-month serum ACE concentrations, ACE2 activity as well as ACE/ACE2 ratios variably, positively correlated with disease duration, ESR, CRP and RF. There were no major differences between the 5 mg bid and 10 mg bid dose groups or between treatmen-naïve and post-washout patients.

Here we only determined ACE concentrations and ACE2 activity. We did not measure ACE activity as some patients were treated with ACE inhibitors (Table 1), which might interfere with this parameter (15, 17). On the other hand, ACE inhibitors to not influence ACE2 activity (17).

Upon JAK inhibition, ACE concentrations significantly increased at 6 and 12 months of treatment compared to baseline. Tofacitinib therapy only transiently increased ACE2 activity at 6 months compared to baseline. Then ACE2 activity returned to baseline level. We could not easily compare our data to those reported by others as there have been major methodological distinctions between studies of others and ours. Nevertheless, in our previous study, 1 year TNF-α inhibitor treatment increased ACE levels and ACE/ACE2 ratio in RA patients similarly to our data presented here (29). Moreover, others also reported increased synovial fluid ACE levels (25, 26), as well as decreased serum ACE2 (24) or unchanged serum ACE levels in RA (25). Collectively, these data suggest that changes in ACE and ACE2 activities might be coordinated in the serum and of the synovium during the course of RA. Our present data extend these observations by illustrating JAK inhibition as a mechanism augmenting serum ACE/ACE2 ratio while tempering RA progression. Hypothetically this can also affect ACE/ACE2 ratio at the tissue level as well. Ours was an uncontrolled, prospective study comparing post-and pre-treatment serum ACE concentration, ACE2 activity and ACE/ACE2 ratio. Hence, it is to be stressed that future prospective studies on the effects of JAKi of ACE and ACE2 distribution between plasma and synovia are required to verify further potential outlooks. With respect to other immune-mediated inflammatory diseases, Potdar et al. (19) found increased expression of ACE2 in active ulcerative colitis (UC) and low small bowel expression of ACE in Crohn’s disease (CD) compared to controls. Thus, although we did not perform any synovial tissue expression studies, increased serum ACE concentrations and unchanged ACE2 activity upon 12-month tofacitinib therapy might indeed reflect antiparallel changes in ACE and ACE2 levels for tissues and serum (17, 24–26). Moreover, increased synovial fluid ACE concentrations have been found in RA in comparison to controls (25, 26, 38). Considering the possible redistribution patterns of ACE and ACE2 mentioned above (17), our results showing an increased ACE/ACE2 ratio upon JAKi therapy might implicate a decreased synovial ACE/ACE2 ratio.

Baseline was a pre-treatment time point when RA patients had high degree of inflammation and disease activity. After 1 year of treatment, as tofacitinib therapy was clinically effective, most RA patients reached the state of clinical remission or low disease activity (LDA). We found it important to seek for correlations between ACE levels, ACE2 activity or ACE/ACE2 ratio and other clinical, laboratory and vascular ultrasonography parameters at baseline, as well as after 6 and 12 months of JAK inhibition. In the regression analyses, baseline ACE concentration or ACE2 activity correlated with ESR, RF and disease duration. On the other hand, after one-year treatment ACE and ACE2 only correlated with RF at various time points and no longer with ESR. Six-month ACE/ACE2 ratio correlated with baseline RF levels. Thus, in JAKi-treated RA patients exerting low-grade inflammation, ACE levels might serve as a marker of autoimmunity (RF). In addition, high baseline RF levels might predict ACE concentration, ACE2 activity and ACE/ACE2 ratio after 6 and 12 months of treatment. Increased plasma ACE levels were reported by some investigators (23), while others determined similar ACE concentrations in RA patients and healthy controls (25).

In the RM-ANOVA analysis, change of ACE levels over time was positively associated with baseline ACPA positivity together with the effects of tofacitinib therapy. Similarly, change of ACE2 activity over time correlated with RF concentrations along with treatment. Thus, in RA, baseline ACPA positivity might be the most important determinant of ACE level changes, while higher RF levels at baseline would determine changes in ACE2 activity over time. These results suggest the importance of autoantibodies in the effects of JAKi on the ACE-ACE2 system.

Our study might have some strengths and limitations. One of the strengths of the present study is that this might be the very first one on the effects of a JAKi on ACE levels and ACE2 activity in RA. Moreover, this study might be the first to evaluate ACE and ACE2 in relation to multiple other biomarkers in JAKi-treated RA patients. Limitations might include the lack of controls, as well as the relatively low number of patients.

## Conclusion

JAK inhibition might increase ACE levels and, transiently, also ACE2 activity in serum samples of RA patients suggesting the shedding and differential redistribution of these RAAS components between the synovium and the blood. Baseline ACE and ACE2 might be associated with disease duration, markers of inflammation (ESR), and autoimmunity (RF). On the other hand, autoantibodies (RF, ACPA) at baseline might be the major denominator of the effects of tofacitinib on the RAAS. The effects of JAK inhibition on ACE levels and ACE2 activity might reflect, in part, the beneficial effects of targeted synthetic drugs on cardiovascular pathology.

## Data availability statement

The raw data supporting the conclusions of this article will be made available by the authors, without undue reservation.

## Ethics statement

The studies involving humans were approved by the Hungarian Scientific Research Council Ethical Committee (approval No. 56953-0/2015-EKL). Written informed consent was obtained from each patient and assessments were carried out according to the Declaration of Helsinki and its amendments. The studies were conducted in accordance with the local legislation and institutional requirements. The participants provided their written informed consent to participate in this study.

## Author contributions

ZS, DK, MF, and ZoP: conceptualization and study organization. ZS, MF, ZoP, and GS: supervision. ZS, DK, MF, KH, and GS: manuscript drafting and finalization. AHo, EV, BS, AHa, ZsP, NB, and SS: patient recruitment, patient examination, and data curation. KH: statistical analysis. AP, MC, MF, DK, AS, and GK: laboratory and imaging assessments, data curation. All authors contributed to the article and approved the submitted version.
